# POF-Based Solar Concentrators Incorporating Dyes and Europium Chelates

**DOI:** 10.3390/ma14102667

**Published:** 2021-05-19

**Authors:** Ander Vieira, Jon Arrue, Begoña García-Ramiro, Felipe Jiménez, María Asunción Illarramendi, Joseba Zubia

**Affiliations:** 1 Department of Applied Physics, School of Engineering of Bilbao, University of the Basque Country UPV/EHU, 48013 Bilbao, Spain; ander.vieira741@gmail.com (A.V.); ma.illarramendi@ehu.eus (M.A.I.); 2 Department of Communications Engineering, School of Engineering of Bilbao, University of the Basque Country UPV/EHU, 48013 Bilbao, Spain; joseba.zubia@ehu.eus; 3 Department of Applied Mathematics, School of Engineering of Bilbao, University of the Basque Country UPV/EHU, 48013 Bilbao, Spain; mariabegona.garciar@ehu.eus (B.G.-R.); felipe.jimenez@ehu.eus (F.J.)

**Keywords:** polymer optical fiber, luminescent solar concentrators, organic dyes, europium chelates, computational models

## Abstract

In this paper, useful models that enable time-efficient computational analyses of the performance of luminescent solar concentrators (LSCs) are developed and thoroughly described. These LSCs are based on polymer optical fibers codoped with organic dyes and/or europium chelates. The interest in such dopants lies in the availability of new dyes with higher quantum yields and in the photostability and suitable absorption and emission bands of europium chelates. Time-efficiency without compromising accuracy is especially important for the simulation of europium chelates, in which non-radiative energy transfers from the absorbing ligands to the europium ion and vice versa are so fast that the discretization in time, in the absence of some simplifying assumptions, would have to be very fine. Some available experimental results are also included for the sake of comparison.

## 1. Introduction

Luminescent solar concentrators (LSCs) are devices that employ doped planar or cylindrical waveguides to harvest and concentrate direct and diffuse sunlight without the need for any tracking system [1]. Nowadays, LSCs are not only seen as a complement of photovoltaic systems employed to reduce the necessary area of solar cells and, thus, their cost, but also as versatile devices whose capacity of concentrating light can be utilized for diverse applications, such as optical sensors or chemical reactors [2]. Although LSCs made from planar waveguides have traditionally been the focus of attention of researchers, those based on doped polymer optical fibers (POFs) are attracting their interest nowadays [2,3,4,5,6]. These POFs can incorporate one or multiple dopants, including a wide variety of available organic dyes and some metalorganic compounds, among others. The role of the dopant molecules is to absorb as much solar energy as possible, and to reemit it in directions that can be guided along the POF to the fiber ends, where the concentrated light can be used for the desired purpose, e.g., for guiding as much light as possible into a solar cell.

**Figure 1:**
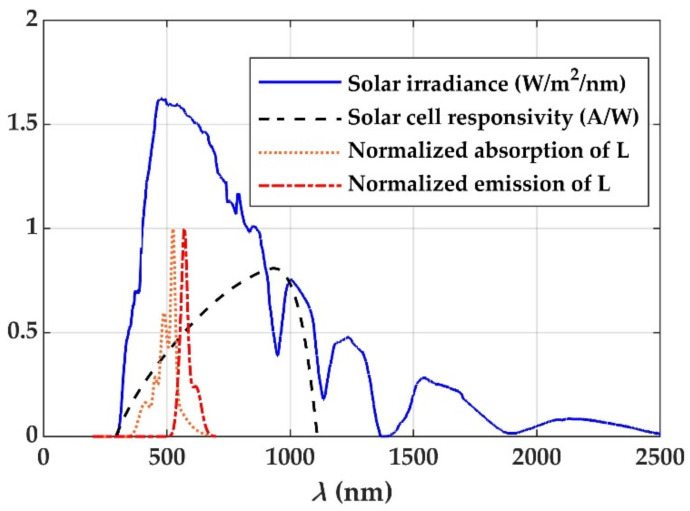
Spectral curve of the terrestrial air-mass-1.5 (AM 1.5) irradiance defined by the ASTM G-173 standard, plotted together with the responsivity curve of a typical solar cell and with the normalized absorption and emission cross sections of the dye Lumogen Orange (L).

The POFs employed as LSCs usually have a uniformly doped thick core (of around 1 mm in diameter, or even thicker) and a much thinner cladding (of around 20 µm, or even no cladding at all) [3,4,7]. The fiber core is typically made of poly (methylmethacrylate) (PMMA). This material presents the advantage of having a relatively low attenuation coefficient (*α_PMMA_*) in the visible spectral region (380–750 nm), which is also the region in which the maximum of the solar irradiance lies and where a great part of the rising edge of the responsivity curve of a silicon solar cell is located (Figure 1). Another advantage of PMMA is that this material has a relatively low glass-transition temperature, which facilitates doping the fiber with suitable organic dyes and metalorganic compounds [4,5,8]. The latter include europium chelates, which are very promising for POF-based LSCs, because they combine a large Stokes shift, which reduces reabsorption losses [4], with the absence of photodegradation of the emitting europium ions. Europium is incorporated in the form of a chelate in order to solve the problem of the low absorption of this metal, thanks to the relatively much higher absorption of the ligands, which transfer the absorbed energy to the europium ion through non-radiative processes [9]. Another important parameter of the dopants employed for LSCs is their quantum yield (ratio between the numbers of emitted and absorbed photons). Nowadays, some new dyes with high quantum yields and/or lower photodegradation (such as the latest Lumogen dyes) have also been employed in LSCs [8,10]. Both types of dopants (europium chelates and dyes) can be mixed with the MMA monomer during the polymerization process of the PMMA [3,4,11], or, alternatively, they can be incorporated into an undoped PMMA POF after its manufacture [12]. An additional advantage of PMMA is its elasticity, which allows for flexible POFs of large diameters, up to several millimeters, to be manufactured, thus facilitating the absorption of the solar energy when the optical fiber is illuminated sideways by the sun.

Due to the recent inclusion of new dopants as suitable candidates for LSCs, the possible combinations of dopants and the design parameters are becoming more and more diverse, since incorporating more than one dopant seems to be the best option [3,13,14]. A single dopant only absorbs a small fraction of the impinging solar spectrum, as shown in Figure 1, which includes the normalized absorption and emission cross sections of Lumogen Orange, one of the dopants investigated for LSCs [4]. If two dyes or a combination of a dye with a europium chelate are employed, it is possible to improve the effectiveness of light-harvesting and the output irradiance [15]. Since the manufacture of new samples of doped POFs in order to measure their behavior can be costly, theoretical models have been utilized to analyze the influence of the most relevant parameters in the early stages of the design and optimization of POF-based LSCs. Furthermore, the complexity of the reported models is constantly increasing, in order to be able to solve new problems or to improve the accuracy of the computational results [7,10,16]. Recently, a model that overcame the characteristic difficulties of methods based on rate equations for the simulation of solar concentrators doped with multiple organic dyes together was reported [7]. This model also incorporated an accurate method for the calculation of the absorbed sun power. However, the model only served for dopants whose energy-level scheme could be treated as two main electronic energy states with vibrational sublevels. It could not be employed for the simulations of other types of dopants, such as europium chelates, in which more than two electronic energy states are involved. Besides, although such model led to reasonably fast calculations for the case of dyes, the reported work did not analyze ways to reduce the computational time. This issue is very important in the case of europium chelates, in which non-radiative energy transfers from the absorbing ligands to the europium ion and vice versa are so fast that the discretization in time would render the calculation of the emission by the europium ion excessively time-consuming. LSCs codoped with this type of dopant have been simulated for the first time in this work, as far as we know.

Specifically, this paper introduces theoretical models that allow the behavior of LSCs codoped with europium chelates and dyes to be described. These models are thoroughly explained and utilized for the attainment of results, which are also compared with some reported measurements. In addition, the computational results of this work allow us to analyze possible ways to improve the performance as LSCs of POFs doped with the most promising combinations of dopants. These include the combination of the europium chelate known as Eu(TTFA)_3_Phen with the dye Lumogen Orange (referred to as Eu/L) or with Coumarin 6 (referred to as Eu/C6) [3,4,17].

## 2. Theoretical Model

### 2.1. Dopants and Energy Levels

**Figure 2:**
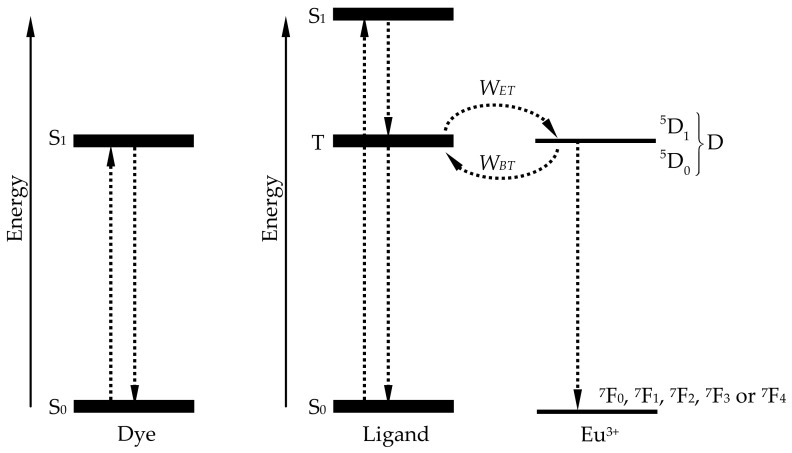
Energy levels of the organic dyes (**left**) and of the europium chelates (**right**).

In this work we develop theoretical models to computationally calculate the behavior of both dyes and rare-earth chelates inside a POF. The luminescent properties of these dopants are dependent on their energy-level structure, which needs to be modeled from the standpoint of the allowed energy transitions and their probabilities. Similarly to the procedure reported in previous works [7,9], we employ a simplified structure of energy levels for each type of dopant (dye or rare-earth chelate), as shown in Figure 2.

In the case of organic dyes, each molecule can transition to a higher electronic energy state (S_1_) by means of the absorption of a photon, from where it can return to the lower electronic energy state (S_0_) emitting a photon (see Figure 2, left) [7]. The quantum yield is close to unity in some dyes, because the rate of radiative spontaneous decays (1/*τ*_r_) is much larger than that non-radiative decays (1/*τ*_nr_), where *τ*_r_ and *τ*_nr_ are the radiative and non-radiative lifetimes.

Rare-earth chelates have a more complex energy-level scheme. Each molecule consists of a single rare-earth ion, such as a europium ion, attached to a few organic ligands. In the case of a typical europium chelate, there are four identical absorbing ligands and a neutral one that does not take part in the absorption. The former can absorb the incoming sunlight in a relatively broad absorption band, and they can transfer the absorbed energy to the europium ion via non-radiative transfer mechanisms. This ion, in turn, emits light in a much narrower spectral band, by means of radiative decays from its excited vibrational energy level to any of its allowed ground vibrational levels. The corresponding energy-level scheme was shown in [9]. Regarding the energy transitions in europium chelates, some simplifying assumptions can be made. In an excited ligand, the energy in the electronic state S_1_ tends to be employed for the excitation of the triplet state T almost instantaneously, so, in practice, S_1_ can be disregarded. This triplet-state energy, in turn, can be transferred to the europium ion. As for the europium ion, its two possible excited vibrational sublevels can be grouped into a single excited sublevel D, due to their relative proximity to each other. This leaves two energy states (T and S_0_) for the ligand, and an excited level D with five allowed sublevels (^7^F_0_, ^7^F_1_, ^7^F_2_, ^7^F_3_ or ^7^F_4_) in the ground energy state for the europium ion (see Figure 2, right).

### 2.2. Rate Equations for Europium Chelates

Three rate equations can be used to describe a POF doped with a europium chelate, which can be written as follows.

Rate equation for the light power emitted by the europium ion:

(1) $$\begin{array}{c} \frac{\partial P^{\pm }(t,z,\lambda_{k})}{\pm \partial z}=\underset{\mathrm{absorption}\;\mathrm{by}\;\mathrm{dopant}}{\underset{︸}{-\sigma^{a}(N-N_{T}(t,z))P^{\pm }(t,z,\lambda_{k})}}\underset{\mathrm{propagation}}{\underset{︸}{\mp \frac{n}{c}\frac{\partial P^{\pm }(t,z,\lambda_{k})}{\partial t}}}-\underset{\mathrm{absorption}\;\mathrm{by}\;\mathrm{host}}{\underset{︸}{\alpha_{PMMA}P^{\pm }(t,z,\lambda_{k})}}\\ +\underset{\mathrm{stimulated}\;\mathrm{emission}}{\underset{︸}{\sigma^{e}(\lambda_{k})N_{D}(t,z)P^{\pm }(t,z,\lambda_{k})}}+\underset{\mathrm{spontaneous}\;\mathrm{emission}}{\underset{︸}{X(\lambda_{k})\;\beta \;W_{sp}^{e}(\lambda_{k})\frac{N_{D}(t,z)}{\tau_{D}}}}. \end{array}$$

Rate equation for the excited population *N_T_* of the ligands in the triplet state:

(2) $$\begin{array}{c} \frac{\partial N_{T}(t,z)}{\partial t}=\underset{\begin{array}{c} \mathrm{rate}\;\mathrm{of}\;\mathrm{increment}\\ \mathrm{caused}\;\mathrm{by}\;\mathrm{sunlight} \end{array}}{\underset{︸}{{Ň}_{sun}}}+\underset{\mathrm{absortion}\;\mathrm{of}\;P}{\underset{︸}{\sum_{k=1}^{M}\frac{\sigma^{a}(\lambda_{k})}{X(\lambda_{k})}P\;(N-N_{T}(t,z))}}\\ +\;\underset{\begin{array}{c} \mathrm{spontaneous}\\ \mathrm{emission} \end{array}}{\underset{︸}{\frac{-N_{T}(t,z)}{\tau_{T}}}}-\underset{\mathrm{energy}\;\mathrm{transfer}}{\underset{︸}{W_{ET}N_{T}(t,z)}}+\underset{\begin{array}{l} \;\mathrm{back}\\ \mathrm{transfer} \end{array}}{\underset{︸}{W_{BT}N_{D}(t,z)}} \end{array}$$

Rate equation for the excited population *N_D_* of the Eu ion in sublevel *D*:

(3) $$\frac{\partial N_{D}(t,z)}{\partial t}=\underset{\begin{array}{c} \mathrm{energy}\\ \mathrm{transfer} \end{array}}{\underset{︸}{W_{ET}N_{T}(t,z)}}-\underset{\mathrm{back}\;\mathrm{transfer}}{\underset{︸}{W_{BT}N_{D}(t,z)}}-\;\underset{\begin{array}{c} \mathrm{spontaneous}\\ \mathrm{emission} \end{array}}{\underset{︸}{\frac{N_{D}(t,z)}{\tau_{D}}}}\;-\underset{\mathrm{stimulated}\;\mathrm{emission}}{\underset{︸}{\sum_{k=1}^{M}\frac{\sigma^{e}(\lambda_{k})}{X(\lambda_{k})}\;P\;N_{D}(t,z)}}.$$

Equation (1) describes the way in which the guided light power varies along the longitudinal axis (*z*) of the optical fiber, which depends on the absorptions and emissions, and on the propagation of light. The ± sign indicates that light can propagate in both directions along the *z* axis. The symbol *P*^±^ is a shorthand for *P*^+^ or *P*^−^, which refer to the light powers propagating in the positive and negative directions, respectively. These light powers are separate magnitudes, which means that Equation (1) needs to be duplicated. Each term on the right-hand side of the equation represents a physical phenomenon, which is indicated below the term. Equation (1) does not describe the behavior of a single wavelength, but rather a continuum of contributions corresponding to all the wavelengths involved. For simulation purposes, the spectrum of light is split into a finite number *M* of wavelengths *λ_k_*, and the system of coupled differential Equations (1)–(3) is solved for each of them.

Equations (2) and (3) describe the evolution of the excited populations, in units of molecules/m^3^, of the absorbing ligand and of the ion (*N_T_* and *N_D_*, respectively) for a given instant *t* and position *z* along the fiber. These concentrations are affected for the most part by the same processes that affect the generated light in its propagation inside the fiber, in addition to being subjected to the influence of the sunlight that illuminates the fiber perpendicularly and is harvested by the solar concentrator. *N_T_* and *N_D_* are also affected by the non-radiative energy transfers from T to D and vice versa, at rates *W_ET_* (s^−1^) and *W_BT_* (s^−1^). The term *X*(*λ*) converts the rate of emissions or absorptions per unit volume (s^−1^ m^−3^) into power per unit distance (W m^−1^), and it can be written as

(4) $$X(\lambda )=\frac{hc\pi {\left(\Phi /2\right)}^{2}}{\lambda }\;\;(J\cdot m^{2}),$$

where Φ is the diameter of the doped fiber core. As for the rest of parameters and physical constants, the notation employed is the same as in [9].

### 2.3. Rate Equations for Organic Dyes

For comparison, let us now consider the rate equations that describe the behavior of a fiber doped with an organic dye. These can be written as follows.

Rate equation for the generated power:

(5) $$\begin{array}{c} \frac{\partial P^{\pm }(t,z,\lambda_{k})}{\pm \partial z}=\underset{\mathrm{absorption}\;\mathrm{by}\;\mathrm{dopant}}{\underset{︸}{-\sigma^{a}(\lambda_{k})(N-N_{2}(t,z))P(\lambda_{k})}}\underset{\mathrm{propagation}}{\underset{︸}{\mp \frac{n}{c}\frac{\partial P^{\pm }(t,z,\lambda_{k})}{\partial t}}}-\underset{\mathrm{absorption}\;\mathrm{by}\;\mathrm{host}}{\underset{︸}{\alpha_{PMMA}(\lambda_{k})P^{\pm }(t,z,\lambda_{k})}}\\ +\underset{\mathrm{stimulated}\;\mathrm{emission}}{\underset{︸}{\sigma^{e}(\lambda_{k})N_{2}(t,z)P^{\pm }(t,z,\lambda_{k})}}\;+\underset{\mathrm{spontaneous}\;\mathrm{emission}}{\underset{︸}{X(\lambda_{k})\beta (\lambda_{k})W_{sp}^{e}(\lambda_{k})\frac{N_{2}(t,z)}{\tau_{r}}}}. \end{array}$$

Rate equation for the excited population *N*_2_:

(6) $$\begin{array}{c} \frac{\partial N_{2}(t,z)}{\partial t}=\underset{\begin{array}{c} \mathrm{rate}\;\mathrm{of}\;\mathrm{increment}\\ \mathrm{caused}\;\mathrm{by}\;\mathrm{sunlight} \end{array}}{\underset{︸}{{Ň}_{sun}}}\;+\underset{\mathrm{absortion}\;\mathrm{of}\;P}{\underset{︸}{\sum_{k=1}^{M}\frac{\sigma^{a}(\lambda_{k})}{X(\lambda_{k})}\;P(\lambda_{k})\;(N-N_{2}(t,z))}}-\;\underset{\begin{array}{c} \mathrm{spontaneous}\;\\ \mathrm{emission} \end{array}}{\underset{︸}{\frac{N_{2}(t,z)}{\tau_{r}}}}\\ -\underset{\begin{array}{c} \mathrm{non}\text{-}\mathrm{radiative}\;\\ \mathrm{decay} \end{array}}{\underset{︸}{\frac{N_{2}(t,z)}{\tau_{nr}}}}\;-\underset{\mathrm{stimulated}\;\mathrm{emission}}{\underset{︸}{\sum_{k=1}^{M}\frac{\sigma^{e}(\lambda_{k})}{X(\lambda_{k})}\;P(\lambda_{k})N_{2}(t,z)}}. \end{array}$$

In Equations (5) and (6), in contrast to what happens in the case of rare-earth chelates, there is only one excited energy state to be considered for the excited molecules, whose concentration per unit volume is *N*_2_. The methods used for the simulation of europium chelates are modified versions of those employed for simulating dyes, which facilitates the simulation of fibers codoped with dopants of both types simultaneously.

In Equations (2) and (6), the term *Ň_sun_* is used to describe the effect of the sunlight on the doped fiber. It represents the rate of increment in *N*_T_ (in Equation (2)) or in *N*_2_ (in Equation (6)) caused by the absorption of the impinging sunlight by the dopant. The calculation of the exact amount of absorbed sunlight was thoroughly described in [7] and it can be done in multiple ways, including algebraic methods and ray-tracing methods. In general, this term can be expressed as follows:

(7) $${Ň}_{sun}=\sum_{k=1}^{M}\frac{I_{sol}\;(\lambda_{k})\Delta \lambda \Phi \eta_{dopant}\;(\lambda_{k})}{X(\lambda_{k})}{\;(\mathrm{molecules}/s/m}^{3})$$

where *I_sol_* denotes the irradiance corresponding to each of the small portions considered for the spectrum of the incoming sunlight, which is sampled into a finite number of wavelengths *λ_k_*. The spectral irradiance that is assumed for the calculations is shown in Figure 1. The parameter *η_dopant_* is the fraction of impinging sunlight that is absorbed by the dopant.

The simulation algorithms presented in this work are based on the numerical resolution of the rate Equation (1) through (3) in the case of europium chelates, and (5) and (6) in the case of dyes.

### 2.4. FDM-Based Simulation

The first approach used for the simulation of fibers doped with europium chelates is applying a finite-difference method (FDM) to Equations (1)–(3) directly. For this purpose, suitable finite intervals Δ*t* and Δ*z* must be chosen to ensure the convergence of the method. Using these intervals, the *t* and *z* axes are sampled into the discrete indices *i* and *j*, while *k* is used to index wavelengths. In accordance to the discretization of *t*, *z* and *λ*, Equations (1)–(3) are approximated as finite-difference equations. A possible way to do so is shown in Equations (8)–(10) for the case of the propagation in the positive direction. These equations are readily generalizable to the negative direction.

(8) $$\begin{array}{r} \frac{P(i+1,j+1,k)-P(i+1,j,k)}{\Delta z}=\frac{-n}{c}\frac{P(i+1,j,k)-P(i,j,k)}{\Delta t}-\alpha_{PMMA}P(i+1,j,k)\\ -\sigma^{a}(k)P(i+1,j,k)\left(N-N_{T}(i,j)\right)+\sigma^{e}(k)P(i+1,j,k)N_{D}(i,j)\\ +X(k)\;\beta \;W_{sp}^{e}(k)\frac{N_{D}(i,j)}{\tau_{D}} \end{array}$$

(9) $$\begin{array}{r} \frac{N_{T}(i+1,j)-N_{T}(i,j)}{\Delta t}={Ň}_{sun}+\sum_{k=1}^{M}\frac{\sigma^{a}\;(k)\;P(i,j,k)}{X(k)}\left(N-N_{T}(i,j)\right)\\ +W_{BT}N_{D}(i,j)-W_{ET}N_{T}(i,j)-\frac{N_{T}(i,j)}{\tau_{T}}, \end{array}$$

(10) $$\begin{array}{ll} \frac{N_{D}(i+1,j)-N_{D}(i,j)}{\Delta t}= & W_{ET}N_{T}(i,j)-W_{BT}N_{D}(i,j)-\frac{N_{D}(i,j)}{\tau_{D}}\\ & -\sum_{k=1}^{M}\frac{\sigma^{e}\;(k)\;P(i,j,k)}{X(k)}N_{D}(i,j). \end{array}$$

Equations (8)–(10) can be solved computationally by iterating over the *i*, *j* and *k* indices, starting from predefined initial values and boundary conditions. For the purposes of simulating a solar concentrator, what is relevant is the behavior over comparatively very long periods (compared to the time scale of the luminescence processes described) and, as such, the iterations over the *i* index are performed until a convergence criterion is satisfied (e.g., that the relative change in the output power from one iteration to the next one be smaller than 10^−8^).

When attempting to use Equations (8)–(10) for practical uses, some simulations are likely to require a prohibitive amount of computational resources and an excessive amount of time. This is due to the nature of the processes described by the equations. Specifically, this issue arises in the case of europium chelates due to the energy-transfer processes between the energy states T and D. These processes happen at a rate that is several orders of magnitude higher than that of the other processes, such as the spontaneous decay of the europium ion. This fact leads to having to choose the value of the interval Δ*t* in the picosecond range. However, since the europium ion’s spontaneous emissions happen on a scale of milliseconds, the number of iterations required over the time axis is significantly higher than that necessary to simulate dye-doped fibers. This problem of practicality leads to the approach explained in the following paragraphs.

To solve Equations (1)–(3) in a more practical manner, one may first consider what happens to Equations (2) and (3) on a time scale that is small enough for any process other than energy transfers between energy levels to be negligible. By removing these terms, we can write the equations as:

(11) $$\frac{\partial N_{T}}{\partial t}\approx -W_{ET}N_{T}(t,z)+W_{BT}N_{D}\;(\frac{1}{\tau_{T}}\;<<\;W_{ET},W_{BT}),$$

(12) $$\frac{\partial N_{D}}{\partial t}\approx W_{ET}N_{T}-W_{BT}N_{D}\;\;(\frac{1}{\tau_{D}}\;<<\;W_{ET},W_{BT}).$$

Equations (11) and (12) form a system of linear differential equations on the unknowns *N_T_*(*t*,*z*) and *N_D_*(*t*,*z*). The exact solution to this approximate system is a linear combination of decreasing exponential functions. In addition, the eigenvalues of the system can be determined to be 0 and −(*W_ET_* + *W_BT_*). Both eigenvalues are less than or equal to zero, which implies that the system will converge into a stable state as *t* increases. The stable solution can be determined by making the derivatives with respect to time equal to zero. This condition leads to the following approximation:

(13) $$W_{ET}N_{T}=W_{BT}N_{D}.$$

Equation (13) can be interpreted as follows: the energy-transfer mechanisms between the T and D energy levels cause *N_T_* and *N_D_* to reach a pseudo-equilibrium state in which they are proportional to each other. If changes on *N_T_* and *N_D_* that are not caused by *W_ET_* and *W_BT_* are negligible in the short time intervals considered, as is the case of the simulations presented here, *N_T_* and *N_D_* can be assumed to be proportional to one another (regardless of the absolute values of *N_T_* and *N_D_*, which can change) without causing significant error in the results.

In order to remove the effect of rapidly varying energy-transfer mechanisms on the computation time, an additional substitution is made:

(14) $$N_{ex}=N_{T}+N_{D}.$$

While the concentration *N_ex_* has no physical meaning in itself, it is useful to use it as an auxiliary variable in a new rate equation that is obtained if Equations (2) and (3) are added:

(15) $$\frac{\partial N_{ex}}{\partial t}=\frac{\partial N_{T}}{\partial t}+\frac{\partial N_{D}}{\partial t}={Ň}_{sun}\;+\;\sum_{k=1}^{M}\frac{\sigma^{a}\;(\lambda_{k})\;P(\lambda_{k})}{X(\lambda_{k})}(N-N_{T})-\frac{N_{T}}{\tau_{T}}-\frac{N_{D}}{\tau_{D}}-\;\sum_{k=1}^{M}\frac{\sigma^{e}\;(\lambda_{k})\;P(\lambda_{k})}{X(\lambda_{k})}N_{D}.$$

In this rate equation, it should be noted that the terms corresponding to the fast energy-transfer mechanisms are not present. In addition, using Equation (13), one can express both *N_T_* and *N_D_* as functions of *N_ex_*:

(16) $$N_{T}=\frac{W_{BT}}{W_{ET}+W_{BT}}N_{ex},\;$$

(17) $$N_{D}=\frac{W_{ET}}{W_{ET}+W_{BT}}N_{ex}.$$

Although Equations (16) and (17) provide the relationships between *N_T_*, *N_D_* and *N_ex_* in very short time scales, these can be assumed to still hold in long time scales, because the slow-varying terms in Equations (2) and (3) are still very small with respect to the fast energy-transfer terms. The assumption that Equation (13) still holds was found to be acceptable by checking it numerically (the quotients *N_D_*/*N_T_* without simplifying assumptions differing in less than 0.0005% from the value $W_{ET}/W_{BT}$ for the LSCs analyzed in this paper). Substituting Eqations (16) and (17) into Equations (1)–(3) results in the following set of rate equations:

(18) $$\begin{array}{ll} \frac{\partial P^{\pm }(t,z,\lambda_{k})}{\pm \partial z}= & -\sigma^{a}(\lambda_{k})P^{\pm }(t,z,\lambda_{k})\left(N-\frac{W_{ET}}{W_{ET}+W_{BT}}N_{ex}\right)\mp \frac{n}{c}\frac{\partial P^{\pm }(t,z,\lambda_{k})}{\partial t}\\ & -\alpha_{PMMA}P^{\pm }(t,z,\lambda_{k})+\sigma^{e}(\lambda_{k})P^{\pm }(t,z,\lambda_{k})\frac{W_{ET}}{W_{ET}+W_{BT}}N_{ex}+X(\lambda_{k})\beta (\lambda_{k})W_{sp}^{e}(\lambda_{k})\frac{W_{ET}}{W_{ET}+W_{BT}}\frac{N_{ex}}{\tau_{D}}, \end{array}$$

(19) $$\begin{array}{ll} \frac{\partial N_{ex}}{\partial t}= & {Ň}_{sun}-\frac{W_{BT}}{W_{ET}+W_{BT}}\frac{N_{ex}}{\tau_{T}}-\frac{W_{ET}}{W_{ET}+W_{BT}}\frac{N_{ex}}{\tau_{D}}-\sum_{k=1}^{M}\frac{\sigma^{e}(\lambda_{k})P(\lambda_{k})}{X(\lambda_{k})}\frac{W_{ET}}{W_{ET}+W_{BT}}N_{ex}\\ & +\sum_{k=1}^{M}\frac{\sigma^{a}(\lambda_{k})P(\lambda_{k})}{X(\lambda_{k})}\left(N-\frac{W_{ET}}{W_{ET}+W_{BT}}N_{ex}\right) \end{array}$$

Equations (18) and (19) could now be solved numerically using a finite-difference method, proceeding in a similar way as with Equations (8)–(10). However, these equations can be solved using an interval Δ*t* wider than the one needed previously without causing convergence problems, thus improving the performance significantly. Since this method involves the use of an approximation, care should be taken to ensure that the conditions are similar to those assumed here, i.e., that the derivative with respect to time of *N_ex_*, which does not contain the fast terms, is much smaller than the fast energy-transfer terms, as follows:

(20) $$\frac{\partial N_{ex}}{\partial t}=\frac{\partial N_{T}}{\partial t}+\frac{\partial N_{D}}{\partial t}\ll W_{ET}N_{T}+W_{BT}N_{D}.$$

This condition can be continually verified by the program as the approximate values of the variables involved are calculated.

### 2.5. Stationary-State Simulation

The previous method for solving the rate equations applies finite differences to both the time *t* axis and the longitudinal *z* axis. Using that method, it can be verified that the solution to the rate equations asymptotically approaches values that are constant with respect to *t*. This fact is consistent with experimental observations. Furthermore, this asymptotic behavior happens at a time scale on the order of the radiative decay lifetime of the dopants used. Since the results of LSCs are measured during time intervals that are much longer than the lifetimes of the dopants utilized in this work, it is the stationary state of the fiber that has to be analyzed. Possible changes in the solar irradiance happen over long time scales, e.g., of seconds or longer, so it can be treated as constant.

If only the stationary state of the solution of the rate equations is needed, it would be desirable to calculate it directly. Equations (1)–(3) can be modified for that purpose by making all derivatives with respect to time equal to zero. This modification removes the *t* variable entirely, assuming that all other terms in the equations are not dependent on time. Therefore, the new equations for rare-earth chelates can be simplified as follows:

(21) $$\begin{array}{ll} \frac{\partial P(t,z,\lambda_{k})}{\partial z}= & -\sigma^{a}\;(\lambda_{k})P(t,z,\lambda_{k})(N-N_{T})-\alpha_{PMMA}\;(\lambda_{k})\;P(t,z,\lambda_{k})\\ & +\sigma^{e}\;(\lambda_{k})P(t,z,\lambda_{k})N_{D}+X(\lambda_{k})\beta (\lambda_{k})W_{sp}^{e}(\lambda_{k})\frac{N_{D}}{\tau_{D}}, \end{array}$$

(22) $$\begin{array}{ll} \frac{\partial P(t,z,\lambda_{k})}{\partial z}= & -\sigma^{a}\;(\lambda_{k})P(t,z,\lambda_{k})(N-N_{T})-\alpha_{PMMA}\;(\lambda_{k})\;P(t,z,\lambda_{k})\\ & +\sigma^{e}\;(\lambda_{k})P(t,z,\lambda_{k})N_{D}+X(\lambda_{k})\beta (\lambda_{k})W_{sp}^{e}(\lambda_{k})\frac{N_{D}}{\tau_{D}} \end{array}$$

(23) $$0=W_{ET}N_{T}-W_{BT}N_{D}-\frac{N_{D}}{\tau_{D}}-\sum_{k=1}^{M}\frac{\sigma^{e}\;(\lambda_{k})P(\lambda_{k})}{X(\lambda_{k})}\;N_{D},$$

and, for organic dyes, the equations become:

(24) $$\begin{array}{ll} \frac{\partial P(t,z,\lambda_{k})}{\partial z}= & -\sigma^{a}\;(\lambda_{k})P(t,z,\lambda_{k})(N-N_{2})-\alpha_{PMMA}\;(\lambda_{k})\;P(t,z,\lambda_{k})\\ & +\sigma^{e}\;(\lambda_{k})P(t,z,\lambda_{k})N_{2}+X(\lambda_{k})\beta (\lambda_{k})W_{sp}^{e}(\lambda_{k})\frac{N_{2}}{\tau }, \end{array}$$

(25) $$0={Ň}_{sun}+\sum_{k=1}^{M}\frac{\sigma^{a}\;(\lambda_{k})P(\lambda_{k})}{X(\lambda_{k})}\;(N-N_{2})-\frac{N_{2}}{\tau_{rad}}-\frac{N_{2}}{\tau_{nr}}-\sum_{k=1}^{M}\frac{\sigma^{e}\;(\lambda_{k})P(\lambda_{k})}{X(\lambda_{k})}\;N_{2}.$$

Note that (22), (23) and (25) are not differential equations. This means that they can be solved directly to obtain values for *N_T_*, *N_D_* and *N*_2_, albeit with a dependence on *P*.

The following substitutions are used to simplify Equations (22) and (23):

(26) $$W_{a}=\sum_{k=1}^{M}\frac{\sigma^{a}\;(\lambda_{k})P(\lambda_{k})}{X(\lambda_{k})},\;W_{e}=\sum_{k=1}^{M}\frac{\sigma^{e}\;(\lambda_{k})P(\lambda_{k})}{X(\lambda_{k})},\;W_{T}=\frac{1}{\tau_{T}},\;W_{D}=\frac{1}{\tau_{D}}.$$

The solution to Equations (22) and (23) is, in matrix form:

(27) $$\left(\begin{array}{c} N_{T}\\ N_{D} \end{array}\right)={\left(\begin{array}{cc} W_{a}+W_{ET}+W_{T} & -W_{BT}\\ -W_{BT} & W_{e}+W_{BT}+W_{D} \end{array}\right)}^{-1}\left(\begin{array}{c} {Ň}_{sun}+W_{a}N\\ 0 \end{array}\right).$$

To simplify (25), the following new auxiliary variables are used:

(28) $$W_{rad}=\frac{1}{\tau_{rad}},\;W_{nr}=\frac{1}{\tau_{nr}},$$

and the solution is analogous to (27):

(29) $$N_{2}={\left(W_{a}+W_{e}+W_{rad}+W_{nr}\right)}^{-1}\left({Ň}_{sun}+W_{a}N\right).$$

Using (27) and (29), the previous differential equations, (21) and (24), can be solved numerically using finite differences over the *z* axis. However, a problem arises due to the powers propagating in both directions (*P*^+^ and *P*^−^), which are calculated by employing the same rate equations. These magnitudes are not independent of each other, due to the fact that their respective effects on *N_T_*, *N_D_* and *N*_2_ affect the opposite direction as well. This means that finite differences cannot be directly applied.

This problem is solved by utilizing an iterative approach. Rather than computing the output powers *P*^+^ and *P*^−^ in a single run through the fiber, they are repeatedly calculated, using the values from the previous iteration in each calculation. The values for *N_T_*, *N_D_* and *N*_2_ are calculated first by using (27) and (29), and the values obtained are used to solve (21) and (24) by using finite differences in both directions in each iteration.

**Figure 3:**
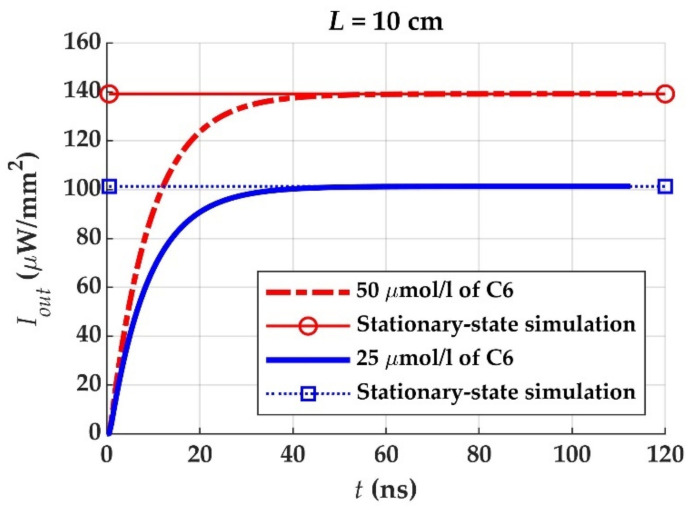
Convergence between the FDM-based simulation and the stationary-state simulation for two different POFs of 10 cm in length whose cores are doped with the dye Coumarin 6, with concentrations of 50 µm/L and 25 µm/L.

Both the FDM-based simulation and the stationary-state one should lead to identical results. This can be checked and observed in Figure 3, which shows that the results obtained with both methods converge to the same value when the elapsed time is long enough for the stationary state to be reached. The results correspond to the output irradiance of two different POFs of 10 cm in length whose core diameter is 980 μm and whose cladding thickness is 20 µm. The cores are doped with the dye Coumarin 6, with concentrations of 50 µm/L and 25 µm/L. The great advantage of the stationary-state method as compared to the other one is the much shorter simulation times required: the respective simulation times for any of the two final values of Figure 3 were 0.6 s and 60 s in our simulations (approximately 100 times faster with the stationary-state method than with the other one). The software used for all the calculations in this work was Matlab^®^ 2019b.

Finally, in order to simulate fibers codoped with both europium chelates and organic dyes, Equations (21) and (24) are combined into a single rate equation. Since the dopants are assumed not to interact directly with each other, Equations (27) and (29) need not be modified. This lack of interaction means that no energy-transfer mechanisms between dopants are considered.

On another front, the mathematical complexity of the rate equations can be further reduced in order to obtain a very rough indication of the influence of some design parameters on the output power. This mathematical reduction was reported in [3], in which a very simple qualitative formula was derived from a rate equation similar to (5), but considering only two wavelengths (*λ_s_* for the emission and *λ_p_* for the absorption). It was also assumed that the average number of photons emitted per unit time and unit volume (*N*_2_ / *τ*) is equal to the average number of photons absorbed per unit time and unit volume, which, in turn, is proportional to the impinging irradiance (*I*_0_) and approximately proportional to [1 − exp(−*α*(*λ_p_*) Φ)], where *α*(*λ_p_*) stands for the product *σ^a^*(*λ_p_*) (*N* − *N*_2_). With all these assumptions, one can readily obtain the simplified qualitative equation for the output power *P_out_* derived in [3], which is:

(30) $$P_{out}\propto I_{o}\Phi \left(\frac{\lambda_{p}}{\lambda_{s}}\right)\frac{W_{sp}^{e}\;(\lambda_{s})}{\alpha (\lambda_{p})}\beta \left(1-e^{-\alpha (\lambda_{p})\;\Phi }\right)\left(1-e^{-\alpha (\lambda_{s})\;L}\right).$$

This equation will be referred to in a discussion about the dependence of the output power on the fiber diameter in Section 3.

## 3. Results and Discussion

In this section, several measurements reported in the literature are compared with the corresponding computational results in order to validate the models for dyes and for europium chelates detailed in Section 2. Such measurements were performed using POFs doped with the combinations of dopants mentioned in the introduction (Eu/L or Eu/C6) [3]. Afterwards, additional simulations serve to reach some conclusions.

### 3.1. Charateristics of the Dopants Employed

**Figure 4:**
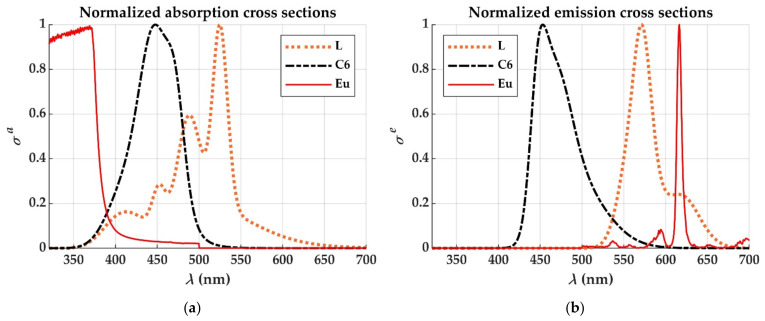
Normalized absorption (**a**) and emission (**b**) cross sections of the dopants employed.

Table 1 shows the absolute values of the parameters needed for the simulation of the dopants considered in this paper. The data were taken or calculated from some reported results [3,4,17,18,19]. The spectral shapes of the absorption and emission cross sections of each of the dopants have been plotted in Figure 4. The concentrations of these dopants in the available samples are shown in the last column of Table 1.

**Figure 5:**
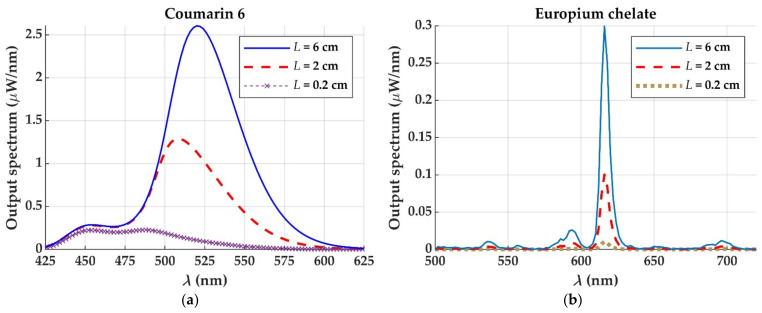
Evolution of the output spectrum with fiber length at the end of an illuminated POF of 1 mm of diameter doped with a concentration of 49 µm/L, either (**a**) of the dye Coumarin 6, or (**b**) of the europium chelate.

Although the europium chelate has much lower cross sections than the two dyes considered (Table 1), the chelate has a potential advantage that is not present in the case of the dyes, which is the absence of overlap between its emission and absorption cross sections (apart from its greater photostability). In a dye-doped POF, the spectrum of the fluorescence obtained from a POF sample of only a few centimeters is strongly influenced by the fiber length (Figure 5a), due to the overlap that occurs between the absorption and emission cross sections. This implies that a great part of the light emitted from any of the dye molecules is reabsorbed in a short fiber length, which increases the losses and reduces the saturation length of the LSC. In contrast, there is no red shift in the spectrum emitted from an Eu chelate-doped POF (Figure 5b), which is a consequence of the absence of overlap between the emission and absorption cross sections. This means that the saturation length is only influenced by the attenuation of the host material, which implies longer saturation lengths, as will be shown below in Section 3.3.

### 3.2. Comparisons between Theoretical and Experimental Results

**Figure 6:**
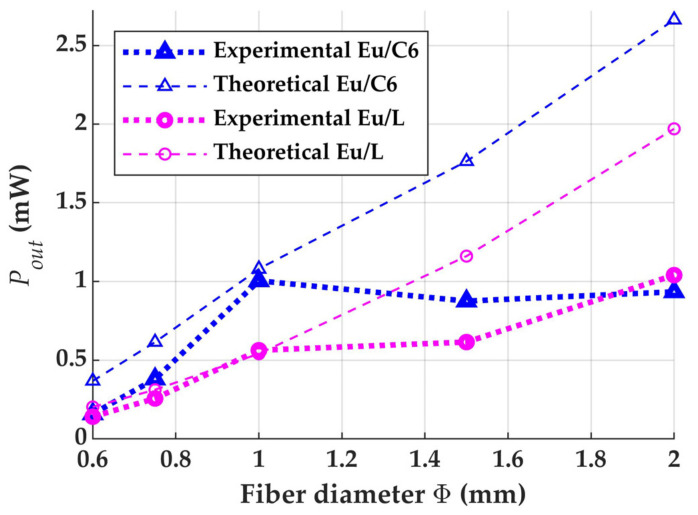
Output powers against POF diameter measured and calculated with the combinations of dopants Eu/C6 and Eu/L, when the illuminated POF lengths are the saturation ones employed in [4].

Figure 6 shows the experimental and computational output powers corresponding to POFs of several diameters doped either with Eu/C6 or with Eu/L when the fiber lengths are the saturation ones employed in [4]. In the case of the POF doped with Eu/L, both experiments and calculations show that the output power increases as the fiber diameter is increased. This behavior can be explained from the greater area illuminated by the impinging sun intensity, this area being the product of fiber length and fiber diameter. Similarly, a positive slope is also predicted theoretically for the POF doped with Eu/C6. The two lower experimental powers for the non-standard diameters of 1.5 mm and 2 mm could be due to several reasons, including physical effects that should be taken into account and experimental uncertainties. Among the former, we have the higher absorption losses when the diameter is larger that could arise as a consequence of the longer paths covered by rays reflected at the cladding-air interface [3]. Among the latter, we have possible differences in the achieved dopant concentration, as well as the differences that could arise from the adjustments that have to be made in the process of drawing the fiber from the preform in order to keep the tension of the drawn fiber constant, which are not the same for POFs of different diameters [20]. Be that as it may, both experimental and theoretical results are reasonably similar, since the differences between them do not seem to be larger than the uncertainties in the experimental results. Moreover, both theoretical and experimental results show that the output powers tend to be larger for the combination Eu/C6 than for the combination Eu/L.

**Figure 7:**
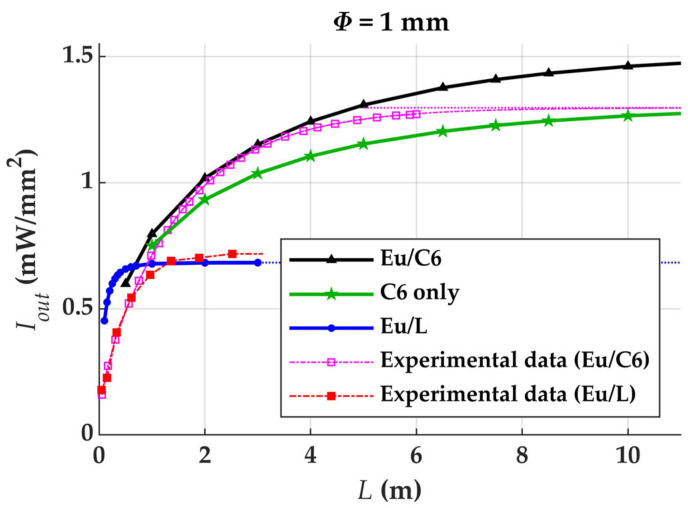
Output irradiances against fiber length for 1 mm POF samples doped with two different combinations of dopants in the concentrations of Table 1, and the corresponding saturation lengths specified at 99.5% of the height of the horizontal asymptote when length *L* tends to infinity.

Figure 7 shows the computational output irradiances against fiber length obtained for the Eu/C6-doped POF, for a POF with the same concentration of C6 but without Eu, and for the Eu/L-doped POF. The figure also includes the experimental curve obtained by measuring Eu/C6 samples of multiple lengths and calculates the best fit curve, which was reported in [4]. In all cases, the POF diameter is 1 mm. The saturation lengths at 99.5% of the height of the horizontal asymptote corresponding to the experimental results are 2 ± 1 m in the case of the Eu/L fiber, and 8 ± 3 m in the case of the Eu/C6 fiber [3,4]. It can be observed that, owing to the rapid saturation of the Eu/L samples, the combination Eu/C6 yields higher output irradiances than the combination Eu/L, except for the shortest lengths considered in the figure. In this sense, a better performance is obtained if Eu/C6 is used instead of Eu/L. Notice that the Eu chelate improves the output irradiance compared to an LSC only doped with C6, and also that the LSC should be codoped with the dye for a high efficiency. Similarly, experimental results perfomed with samples codoped with another organic dye (Coumarin 1) and with the same Eu chelate also corroborate that the chelate serves to further improve the high output irradiance caused by the effect of the dye [4]. Specifically, a sample of 1 mm in diameter doped with 90 µm/L of C1 and with 30 µm/L of Eu (lower concentration of Eu) and illuminated along 6 cm yielded a higher output irradiance (11 µW/mm^2^) than another sample in which the two concentrations where interchanged (6 µW/mm^2^ in this case). Note, also, that the irradiances achieved in the case of Eu/C6 are greater than the direct irradiance of the sun, provided that the fiber length is large enough. These results, together with the fact that the spectrum is shifted towards more suitable wavelengths, shows that the solar concentrator serves to improve the performance of the system.

### 3.3. Europium Chelate for the Improvement of the Output Power

**Figure 8:**
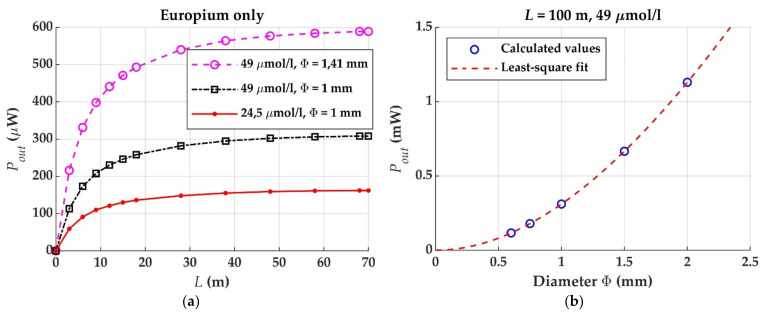
(**a**) Output powers against fiber length for various combinations of Eu concentration and fiber diameter, when the POF sample is only doped with Eu chelate. (**b**) Output powers against fiber diameter corresponding to Eu-doped POFs of 100 m, and least-square fit using Equation (31).

Figure 8a shows that the saturation length is much larger if a fiber is only doped with Eu chelate, irrespective of the dopant concentration and of the fiber diameter. In this case, the saturation is only caused by the attenuation of the host material, owing to the absence of overlap between the absorption and emission cross sections. Figure 8 also illustrates that the output power tends to increase if the concentration, or the length, or the diameter of a doped optical fiber are increased. If the diameter is multiplied by the factor $\sqrt{2}$ or if the concentration is multiplied by 2, the output power nearly doubles. Let us now analyze the dependence in more detail. When the concentration is constant and the fiber length is greater than the saturation one, and using a similar reasoning as in [3], it can be posed that

(31) $$P_{out}(\Phi )\approx P_{max}\;(\Phi )\left(1-e^{-\alpha_{fit}\;\Phi }\right)\approx C_{1}L\;\Phi \left(1-e^{-\alpha_{fit}\;\Phi }\right),$$

where *α_fit_* is a fit parameter that is related to the attenuation of sunlight, Φ is the fiber diameter, *P_max_* stands for the power obtained when *α_fit_* tends to infinity, and the factor (1 – exp(−*α_fit_* Φ)) represents the fraction of sun power absorbed by a fiber cross section of diameter Φ. In order to check the validity of Equation (31), Figure 8b shows the curve of *P_out_*(Φ) calculated computationally without simplifications, together with the least-square fit to Equation (31) for a constant concentration of Eu chelate and for a fiber length of 100 m, which is longer than the saturation length for the concentration considered. The high degree of coincidence between the two curves confirms that Equation (31) is accurate enough for the case of this dopant. In turn, *P_max_* can be assumed to be proportional to the incident area where the sun intensity (W/m^2^) impinges on the fiber surface. *C*_1_ is the proportionality factor. Besides, when the factor (1 – exp(−*α_fit_* Φ)) is no larger than 0.4, it can be replaced by (*A* Φ – *B* Φ^2^) using its Taylor series expansion, with a maximum relative error that is smaller than 5% (*A* and *B* are positive numbers). In such a case, *P_out_*(Φ) is proportional to (*A* Φ^2^ – *B* Φ^3^). Therefore, the output irradiance is proportional to (*A* – *B* Φ), so it decreases linearly with Φ. For the curve of Figure 8b, *α_fit_* = 0.2 mm^−1^, so the maximum diameter in order for this approximation to be valid within an error of 5% is Φ = 2.5 mm.

## 4. Conclusions

This paper provides time-efficient approaches that serve to perform accurate simulations of luminescent solar concentrators based on polymer optical fibers codoped with organic dyes and/or metalorganic compounds such as europium chelates. Our computational models provide quantitative results that agree reasonably well with the experimental results. These models may be a valuable tool for the design of POF-based LSCs, whose prototypes are still at the early stages of investigation, owing to the ever-increasing variety of promising dopants that have been developed in the last few years. The models illustrate how to achieve time efficiency without compromising accuracy, which is especially important for the case of europium chelates, in which non-radiative energy transfers from the absorbing ligands to the europium ion and vice versa are so fast that the discretization in time would have to be very fine if no approximations were made. We also show that adding a europium chelate to the dye can serve to improve the output irradiance. In the case of using only the europium chelate, the saturation fiber lengths are longer, and the output irradiances are smaller than in the case of codoped POFs, but the output irradiance increases, and in a linear way, if the fiber diameter is decreased.

## Figures and Tables

**Table 1:** Parameters of the dopants analyzed in this paper.

Dopant	*σ*^*a*^ (m^2^) at *λ_peak_* of Absorption	*σ*^*e*^ (m^2^) at *λ_peak_* of Emission	Decay Lifetimes and Transition Rates	Concentration in the Available Samples
Eu(TTFA)_3_Phen (Eu)	9.74 × 10^−21^	6.41 × 10^−24^	*τ_T_* = 10^−5^ s *τ_D_* = 10^−3^ s *W_ET_* = 8 × 10^8^ s^−1^ *W_BT_* = 2 × 10^8^ s^−1^	49.2 µm/L (2.961 × 10^22^ molecules/m^3^)
Lumogen Orange (L)	2.38 × 10^−20^	2.52 × 10^−20^	*τ_rad_* = 6 × 10^−9^ s *τ_nr_* = 1.1 × 10^−7^ s	29.5 µm/L (1.777 × 10^22^ molecules/m^3^)
Coumarin 6 (C6)	3.90 × 10^−20^	1.87 × 10^−20^	*τ_rad_* = 2.5 × 10^−9^ s *τ_nr_* = 8.9 × 10^−9^ s	49.2 µm/L (2.961 × 10^22^ molecules/m^3^)

## Data Availability

Not applicable.
